# Domesticating esoteric Buddhism: lay women, Foxin Jing practice, and somatic authority in contemporary China

**DOI:** 10.3389/fsoc.2026.1867040

**Published:** 2026-09-09

**Authors:** Tianyan Yin, Yaoping Liu, Daohua Xu

**Affiliations:** Department of Global Buddhism, Institute of Science Innovation and Culture, Rajamangala University of Technology Krungthep, Bangkok, Thailand

**Keywords:** contemporary Chinese Buddhism, embodied religion, Foxin Jing, heart-of-mind esoteric Buddhism, lay women, lived esotericism, religious agency

## Abstract

This study examines how lay women engage with, adapt, and transmit the heart-mind esoteric Buddhist tradition known as Foxin Jing (佛心经) in contemporary China. Previous scholarship on Chinese esoteric Buddhism has predominantly focused on issues related to legitimacy, lineage, institutional power, and master-disciple relations. However, there has been a relative lack of focus on the role played by women in maintaining esoteric Buddhist practice via the domestic, affective, and embodied dimensions of religion. Drawing on qualitative research with twenty lay women practitioners, this study shows that Foxin Jing is experienced not merely as an esoteric ritual or protective dhāraṇī, but as an embodied heart-mind discipline integrated into domestic routines, interpersonal relationships, and personal cultivation. The findings demonstrate that women sustain the practice through domestic and informal lay settings, relational networks, and bodily-affective experiences involving voice, breath, silence, emotional composure, and changes in bodily tension. The article further develops the concept of somatic authority to explain how embodied transformation becomes socially recognizable as evidence of religious efficacy and a basis for interpersonal trust. These findings show that the continuity of Foxin Jing depends not only on formal institutions and lineage authority, but also on women’s everyday discipline, relational transmission, and embodied religious credibility.

## Introduction

In the Heart-of-Mind (Xinfa 心法) tradition of esoteric Buddhism in mainland China, women participate as followers, practitioners, students, ritual organizers, community supporters, and mediators between esoteric teachings and everyday religious life (Payne, 2022; Xu and Liu, 2025). Female practitioners occupy an increasingly visible position in contemporary Chinese Buddhism through access to practice communities, religious education, devotional networks, and various forms of Buddhist participation (Lai, 2022; Lau, 2022; Lin, 2023; Wang, 2023; Yun, 2023; Geng, 2025). However, greater visibility does not necessarily imply equal access to religious authority. At the same time, ritual authority remains strongly structured through guru-disciple relations and transmission lineages, while women’s contributions are often framed as devotional or supportive rather than as forms of agency that shape the continuity of the tradition itself (Esler, 2020; Xu and Liu, 2025). This tension makes it necessary to move beyond the question of whether women are present and instead examine how their participation becomes religiously consequential within the lived practice of contemporary esoteric Buddhism.

Such a shift is particularly important because transmission and authority in esoteric Buddhism cannot be reduced to texts, doctrines, or institutional succession alone (Bentor and Shahar, 2017; Larson, 2020; Proffitt, 2023). They are also constituted through teacher-disciple relations, ritual repetition, mantra and dhāraṇī practice, bodily discipline, affective experience, and the internalization of teachings in everyday life. In the Heart-of-Mind tradition, transmission therefore operates not only as a vertical passage of authorized knowledge but also as a social and embodied process through which practices are repeated, interpreted, and made meaningful within communities (Xu and Liu, 2025). However, existing studies of Chinese esoteric Buddhism have largely privileged questions of mastery, lineage genealogy, legitimacy, and institutional continuity (Buckelew, 2019; Xu and Liu, 2025), leaving women’s everyday religious practices comparatively underexamined. Scholarship on Chinese Buddhist women likewise suggests that agency does not necessarily require formal leadership or direct resistance to hierarchical structures; structural asymmetry may coexist with women’s capacity to cultivate status, subjectivity, and religious authority from within those structures (Péronnet, 2022). The unresolved issue, therefore, is how such agency is produced through embodied and relational esoteric practice among lay women.

This article addresses that gap by asking: *How do women in the Heart-of-Mind Esoteric Buddhist tradition in contemporary China embody esoteric practice and contribute to religious transmission?* It examines how women understand and experience the Dharma Heart through bodily and affective practice, how they situate themselves within guru-disciple and ritual relations, and how Foxin Jing is sustained through everyday practice and interpersonal transmission. Rather than defining agency primarily through public leadership, doctrinal authority, or opposition to male power, this study approaches female agency as a form of religious work through which esoteric practice becomes sustainable, socially meaningful, and transmissible in ordinary life. In doing so, the article shifts attention from institutional representation to the lived processes through which women participate in the reproduction of Heart-of-Mind Buddhism.

## Literature review

### Female religious agency in contemporary Chinese Buddhism: from institutional visibility to religious practice

Since the reforms initiated under Deng Xiaoping in 1978, Buddhism in mainland China has gradually re-emerged from the severe suppression and institutional disruption of religious life during the Cultural Revolution through temple reconstruction, the reopening of monastic institutions, renewed Buddhist education, and expanding lay participation (Ji, 2009). However, this revival cannot be understood solely through institutional indicators such as temple numbers, sangha size, ordination statistics, or formal organizational structures, because these measures reveal only part of women’s participation in contemporary Buddhist life. Péronnet (2021, 2022) demonstrates that gender asymmetry within Chinese Buddhist institutions does not necessarily translate directly into female subordination, since differentiated structures can also become resources through which women cultivate status, legitimacy, and religious authority. Lau (2022) similarly shows that Chinese nuns and laywomen actively seek practice opportunities, participate in transnational meditation networks, and create alternative spaces of religious cultivation when access to established monastic environments is restricted. Together, these studies shift the analytical focus from women’s numerical presence toward the forms of agency that emerge within particular religious structures, suggesting that women’s participation should be examined not only through institutional position or public leadership, but also through the practices by which religious subjectivity and continuity are produced. This shift is especially important for Heart-of-Mind Esoteric Buddhism, where the embodied and everyday dimensions of lay women’s religious participation remain comparatively underexamined.

### The teacher’s influence: authority and continuity through vertical networks

Esoteric Buddhist traditions are structured through forms of transmission in which teachers, disciples, ritual authorization, and lineage remain central to the production of religious legitimacy (Bentor and Shahar, 2017; Larson, 2020; Proffitt, 2023). In this context, authority is not merely textual or doctrinal but relational, because the guru functions simultaneously as an interpreter of practice, a source of legitimacy, and a mediator of transmission. At the same time, disciples develop religious orientation through sustained participation in these networks. Péronnet’s (2021, 2022) work on Chinese Buddhist women illustrates how teacher–disciple relations can shape institutional trajectories, understandings of orthodoxy, and the reproduction of particular religious practices, while studies of guru authority emphasize the importance of trust, ritual intimacy, discipline, and affective attachment in sustaining religious networks (Phuntsho, 2022; Rigopoulos, 2022; Di Placido, 2023; Parasher, 2016). These vertical networks, however, should not be understood as one-way channels in which authority descends from master to disciple, because religious traditions persist only when authorized teachings are reproduced within communities of practitioners. Studies of spiritual authority further indicate that loyalty to teachers and participation in religious networks can generate trust and influence even in the absence of formal office (Rudert, 2017; Spina, 2017; Mukhopadhyay, 2020; Copeman et al., 2023; DeNapoli, 2023). For Heart-of-Mind Buddhism, Xu and Liu (2025) establish the importance of lineage and authority for legitimacy and continuity. However, a significant analytical question remains: how does authorized teaching become socially reproducible once it enters lay networks and everyday practice?

### From institutional representation to lived esoteric practice

Institutional data provide a useful point of contrast for examining women’s religious participation in Chinese Buddhism. Between 2009 and 2021, women accounted for approximately 27,760 of 70,610 recorded ordinations, or 39.31%, compared with 42,850 male ordinations, or 60.69%, indicating persistent gender asymmetry within formal monastic structures (Péronnet, 2022). However, such figures cannot capture forms of religious participation that occur outside ordained or institutionally recognized settings, especially in lay esoteric traditions where practice may be situated in domestic environments, informal networks, and everyday routines rather than temples or officially organized communities (Welter, 2017; Zhang and Ji, 2018; Schneider, 2023). The distinction between institutional representation and lived practice therefore provides an important conceptual transition for this study: rather than treating women’s formal position within Buddhist institutions as a proxy for religious agency, the analysis focuses on how esoteric practice acquires meaning, credibility, and continuity within lay life. This perspective directs attention to a dimension that remains insufficiently developed in existing scholarship on Heart-of-Mind Buddhism: namely, how Foxin Jing becomes integrated into women’s everyday lives, how its efficacy is experienced and socially recognized, and how such experiences contribute to the circulation of the tradition beyond formal institutional authority.

### From embodied experience to somatic authority

Embodied religion provides an important framework for understanding how religious experience is constituted through bodily sensation, sensory practice, affect, and habitual action rather than through belief or doctrine alone (Csordas, 2002; McGuire, 2008). Lived religion similarly directs attention to how religious meaning is produced within everyday practice outside formal institutions, while studies of ritual embodiment and emotional regulation explain how repeated religious practices shape bodily dispositions and affective responses (McGuire, 2008). These perspectives, however, do not fully explain how embodied change becomes a socially recognizable source of religious credibility. This study therefore uses the term somatic authority to refer to the process through which cultivated bodily and affective dispositions—such as emotional composure, steadiness in recitation, controlled breathing, reduced reactivity, and consistency of practice—are recognized by other practitioners as evidence that a religious practice is efficacious and that its practitioner is worthy of trust. Somatic authority is thus distinct from embodied experience itself: embodiment concerns how religion is lived through the body, whereas somatic authority concerns how embodied transformation acquires relational recognition and becomes consequential for religious credibility and transmission. Its analytical novelty lies in identifying the mechanism through which bodily change moves from an individual experience to a socially meaningful form of informal authority, particularly in contexts where practitioners lack formal office, ordination, or institutional certification. In the empirical material examined here, somatic authority is operationalized through instances in which women evaluate Foxin Jing through observable changes in themselves or others—such as greater calmness, slower emotional reactions, bodily relaxation, or consistency of practice—and subsequently use those changes as grounds for trust, continued practice, or informal transmission.

## Methods

### Research design

This study employed a qualitative hermeneutic phenomenological design to examine how lay women experience, interpret, and embody Foxin Jing within the Heart-of-Mind Esoteric Buddhist tradition in contemporary China. The methodological approach was informed primarily by van Manen’s hermeneutic phenomenology, which understands lived experience as situated meaning that becomes accessible through reflective interpretation rather than as an objective phenomenon to be merely described (van Manen, 2016). Accordingly, Foxin Jing was approached not simply as a ritual text or religious technique, but as a lived practice embedded in participants’ bodily sensations, affective experiences, domestic routines, interpersonal relationships, and religious worlds. Interpretation therefore moved between particular experiential accounts and their broader social and religious contexts, enabling the analysis to examine how women understood and gave meaning to Foxin Jing within everyday life in post-Reform China.

### Field sites and research context

The study conducted multiple-sited qualitative field research on different sites practicing Heart-of-Mind (Xinfa 心法), with Shandong serving as the main research site for investigating this type of religious practice due to its importance in shaping and spreading the practice of Foxin Jing by lay practitioners. These multiple-sited practices include lay practice groups, semi-religious institutions, household settings, and certain temple surroundings, where laywomen practiced, spread, and interpreted Foxin Jing as a part of their daily religious practices. The selection of these field sites was guided by purposive sampling methods, which enabled the researcher to investigate Foxin Jing not only as a ritual but also as a living, embodied practice in a variety of social locations. Limited attention was also given to wider pan-Chinese networks, including relevant connections beyond Shandong, to situate the practice within broader patterns of Heart-of-Mind transmission. The design for multiple sites was not intended to generate comparative information for the region but instead to provide the context necessary to understand how laywomen in present-day China encounter, negotiate, and enact Foxin Jing.

### Participants and sampling strategy

Participants in this study were 20 lay women (Table 1) who practiced Foxin Jing (佛心经/Mahā-Pratisarā Dhāraṇī) within the Heart-of-Mind Esoteric Buddhist Tradition in mainland China. All participants were non-monastic practitioners who did not hold bhiksuni status or formal sangha affiliation and who incorporated Foxin Jing into their everyday religious lives. Inclusion criteria required participants to be women aged 25 years or older, actively engaged in Foxin Jing practice, and able to reflect on the bodily, affective, religious, and social meanings of that practice. Participant recruitment proceeded concurrently with preliminary analysis. Rather than determining sample adequacy through numerical representativeness, the study assessed adequacy through the recurrence, depth, and interpretive sufficiency of experiential accounts. Recruitment continued until additional interviews elaborated existing patterns of domestic practice, relational learning, bodily experience, and affective change without producing a substantially new experiential structure relevant to the research questions. The final sample of 20 participants was therefore considered sufficient for the interpretive aims of the study. Although the participants were geographically concentrated in Shandong, this concentration reflects the study’s purposive focus on a major Heart-of-Mind practice context rather than an attempt to represent all practitioners in mainland China.

**Table 1:** Overview of participants.

Participant code	Age range	Occupation background	Years of Foxin Jing practice	Region
P1	40–45	Housewife	12	Shandong
P2	35–40	Private employee	6	Shandong
P3	50–55	Self-employed	18	Shandong
P4	45–50	Teacher	10	Shandong
P5	30–35	Administrative staff	4	Shandong
P6	55–60	Retired	20+	Shandong
P7	40–45	Housewife	8	Shandong
P8	35–40	Nurse	5	Shandong
P9	45–50	Trader	15	Shandong
P10	50–55	Housewife	22	Shandong
P11	30–35	Freelancer	3	Shandong
P12	40–45	Private employee	9	Shandong
P13	55–60	Retired	25	Shandong
P14	35–40	Small business owner	6	Shandong
P15	45–50	Housewife	14	Shandong
P16	30–35	Service staff	4	Shandong
P17	50–55	Self-employed	16	Shandong
P18	40–45	Teacher	11	Shandong
P19	55–60	Retired	30	Shandong
P20	35–40	Private employee	7	Shandong

The sampling strategy combined maximum variation and snowball sampling, with limited convenience sampling used only for initial field entry. Maximum variation sampling was employed to capture diversity in age, occupation, family circumstances, duration of practice, and practice setting, allowing the study to examine both shared experiential patterns and socially situated variation. Snowball sampling served as the principal recruitment strategy because Foxin Jing practice is largely private, relational, and trust-based, and many practitioners are not formally registered within religious institutions. Initial participants were identified through existing Heart-of-Mind networks, after which additional participants were recruited through chain referrals. This approach facilitated access to information-rich practitioners while respecting the sensitivity of esoteric religious practice. Recruitment was conducted with informed consent, anonymity, and attention to participants’ willingness to disclose personal religious experiences.

### Data collection methods

Data were collected through three complementary techniques: semi-structured in-depth interviews, non-interventional field observations, and reflective field notes. Semi-structured interviews served as the primary source of experiential data and enabled participants to describe how they performed, interpreted, and embodied Foxin Jing. Interviews were conducted in person or online, lasted 45–90 min, were audio-recorded with participants’ consent, and transcribed verbatim. The interview guide was developed in accordance with the research questions and the phenomenological approach, progressing from participants’ religious backgrounds and pathways into Heart-of-Mind practice to more focused questions about bodily experience, affect, religious identity, and the meaning of Foxin Jing. Interviews were conducted in Chinese and subsequently translated into English for analysis and publication; key religious terms and culturally specific expressions were retained in Chinese characters at their first occurrence to preserve their original semantic context. In addition to interviews, the researcher conducted field observations as a non-intrusive method of capturing the parts of Foxin Jing performance that might not be easily expressed verbally, specifically sound, body, and emotion of dhāraṇī recitation, such as the rhythm, pace, repetitiveness, timbre of voice, periods of silence, bodily postures, breathing exercises, facial expressions, and body movements. Finally, reflective field notes written by the researcher immediately after the interviews and observations provided additional contextual information, social processes at play, affective experience, and initial analytical insight.

### Research instrument

The researcher served as the primary instrument for data collection and interpretation through a hermeneutic-phenomenological lens. To ensure methodological coherence and analysis of the richness of data, three additional instruments were used: a semi-structured interview guide, an observation checklist, and field notes. The interview guide was developed from the research questions and designed to facilitate reflection on participants’ embodied experiences of Foxin Jing, beginning with their personal backgrounds and pathways into the Heart-of-Mind tradition and progressing to everyday practice, bodily sensations, affective responses, religious identity, and changes in the meaning and practice of Foxin Jing across their individual practice histories. Observations of Foxin Jing practice were conducted as they were happening in naturalistic settings such as domestic and semi-domestic or community spaces, with a focus on place of performance, rhythm and speed of recitation, voice, silence, body posture, breath, body movements, interpersonal interaction, and affective climate. Field notes were taken immediately after each interview and observation. They reflected the context of the encounter, the interaction itself, impressions, preliminary interpretations, and analytical memos on how the data relate to theorizations of lived religion, embodied practice, gendered subjectivity, and religious transmission. These instruments were used flexibly, allowing the researcher to follow participants’ narratives while maintaining methodological consistency.

## Data analysis

Data were analyzed using hermeneutic phenomenological analysis, with initial coding used only to organize the empirical material. Interview transcripts, field observations, and reflective field notes were read repeatedly to identify significant experiential accounts related to domestic practice, relational transmission, bodily sensations, affective responses, and changes in the meaning of Foxin Jing. Analysis followed a hermeneutic movement between part and whole: specific statements and observed practices were interpreted in relation to each participant’s broader life context and then reconsidered against patterns emerging across the full dataset. Codes were compared across interviews, observations, and field notes to identify recurrent meanings while preserving variations in individual experience. Particular attention was given to bodily descriptions, voice, breathing, silence, emotional responses, interpersonal relationships, and temporal changes in practice. Embodied religion, lived religion, and gender were used as sensitizing frameworks rather than predetermined coding categories. Interpretations were continually reviewed through comparison across data sources and reflective analytical memos. Reflexive memoing was also used to examine how the researcher’s field relationships, assumptions, and interpretive position shaped data production and interpretation. Emerging interpretations were repeatedly checked against interview transcripts, observation records, and participants’ broader contexts to distinguish participants’ accounts from the researcher’s analytical reading. This process generated two overarching empirical patterns: (1) the domestication and informal circulation of Foxin Jing through lay spaces and relational networks, and (2) the learning, adaptation, and embodiment of Foxin Jing through bodily, affective, relational, and temporal experience.

## Results

Analysis of interviews, field observations, and reflective field notes identified two interconnected patterns in women’s experiences of Foxin Jing. First, the practice was sustained through domestic and informal lay settings rather than through fixed institutional structures. Second, women learned, adapted, and interpreted Foxin Jing through bodily, affective, relational, and temporal experience. Although all 20 participants contributed to these patterns, the following sections foreground selected information-rich cases that most clearly illuminate the recurrent meanings identified across the dataset.

### Establishing domestic and lay esoteric spaces for Foxin Jing practice

Across the participants, Foxin Jing practice was organized primarily through everyday lay settings rather than fixed institutional structures. Two recurrent patterns were especially visible: individual practice conducted in domestic or semi-private settings, and flexible practice incorporated into occupational and everyday routines. These settings allowed women to sustain Foxin Jing without depending on formal membership, fixed ritual schedules, or continuous access to temples.

#### The case of P2

P2, a private-sector employee in her late thirties with six years of Foxin Jing practice, emphasized privacy as an important condition for sustaining the practice. Unlike collective Buddhist activities that require scheduled attendance or public participation, she preferred to recite in quiet settings where others did not observe her. She explained:

“It is very tranquil and private. No need to share it with anyone else.”

For P2, privacy did not indicate withdrawal from religious life but created the conditions in which the practice could be maintained alongside work and everyday responsibilities. The absence of a public audience allowed Foxin Jing to become a personal and repeatable discipline, showing how semi-private settings can function as viable spaces for sustained lay esoteric practice.

#### The case of P4

P4, a teacher in her late forties with approximately ten years of Foxin Jing practice, practiced both individually at home and occasionally within small lay groups. Her account shows how the practice was integrated into the interpersonal demands of professional life rather than separated from them. She particularly associated continued practice with changes in how she responded to pressure in relationships with students and colleagues:

“I would not say the problem disappeared, but my reactions have changed. I do not explode as quickly as I used to.”

Rather than requiring withdrawal from work or participation in a fixed religious schedule, Foxin Jing provided P4 with a practice that could be sustained alongside her professional responsibilities. Her experience illustrates how lay esoteric practice becomes embedded within ordinary occupational life while remaining anchored in personal and domestic practice.

#### The case of P8

P8, a nurse in her late thirties with five years of practice, practiced Foxin Jing primarily at home. Her occupational context involved demanding routines and periods of emotional pressure, making the flexibility of private practice particularly important. She described becoming increasingly attentive to changes in her own inner state:

“Now I can sense the signs earlier. If my heart starts to become unstable, I know to pause.”

P8’s account shows that the accessibility of Foxin Jing lay practice lies partly in its capacity to be incorporated into everyday life without requiring a separate institutional setting. For her, practice at home provided a space in which the pressures of working life could be interrupted by a deliberate moment of inward attention.

#### The case of P16

P16, a service-sector worker in her early thirties with four years of Foxin Jing practice, began practicing during a period of work-related exhaustion. Her broader account emphasized the simplicity of Foxin Jing and the fact that it did not require elaborate ritual preparation, which made it practicable within a fast-paced working life. She described its purpose in modest and everyday terms:

“I do not recite Foxin Jing for anything major. I want my family to be safe and my heart to be unwavering.”

Her account is significant because it situates Foxin Jing neither in spectacular esoteric experience nor formal ritual performance, but in ordinary concerns for family stability and personal steadiness. The practice could therefore be maintained privately at home and adapted to the limited time available within contemporary working life.

#### The case of P17

P17, a self-employed woman in her early fifties with approximately sixteen years of Foxin Jing practice, described how her practice had become increasingly home-based and flexible over time. Rather than following a fixed external schedule, she adjusted the pace and timing of recitation to her own condition. As she explained,

“In recent years, I have practised more in this way—at home, slowly, following the state of my heart.”

Her account shows how a long-term practitioner may gradually reorganize Foxin Jing around a more individualized domestic rhythm without abandoning the practice itself.

Taken together, these cases show that Foxin Jing is sustained through two closely related conditions: access to domestic or semi-private practice spaces and the flexibility to incorporate recitation into occupational and everyday routines. These conditions provide the social and spatial basis for the processes of learning, adaptation, transmission, and embodiment examined in the following section.

### Female lay practitioners learning, adapting, and embodying Foxin Jing in everyday life

Learning Foxin Jing within the Heart-of-Mind tradition did not follow a single institutional pathway. Across the participants, entry into the practice occurred through friends, family members, fellow practitioners, and informal lay networks rather than through a standardized monastic curriculum or formal religious program. Women often encountered Foxin Jing at moments when ordinary life had become difficult to manage—during family tension, occupational pressure, illness, emotional exhaustion, or periods of uncertainty—and continued practicing when they experienced changes that they considered meaningful. The practice was therefore learned not simply as a text or doctrinal teaching, but through listening, observing, repeating, and evaluating its effects in everyday life. Across differences in age, occupation, and duration of practice, participants described a gradual process through which Foxin Jing moved from something introduced by another person into a personally meaningful discipline of the heart-mind.

#### The case of P3

P3, a self-employed woman in her early fifties with eighteen years of Foxin Jing practice, encountered the practice through personal relationships rather than through a temple-based program. Over time, she incorporated recitation into the temporal gaps of household life rather than establishing a separate ritual schedule. She explained:

“I do not set aside a specific time like in temples. I normally recite Foxin Jing during my preparation for breakfast or after the children are asleep. The most essential thing is having a peaceful heart.”

For P3, flexibility was not evidence of a weaker or incomplete religious practice. It was precisely what allowed Foxin Jing to remain sustainable alongside everyday responsibilities. Preparing breakfast, waiting until family members were asleep, and using quieter moments at home became part of her practice rhythm. Her account shows that learning Foxin Jing also involved adapting it: domestic time was gradually reorganized as religious time, and repetition became more important than adherence to a fixed institutional schedule.

#### The case of P7

A different pathway was described by P7, a housewife in her mid-forties who had practiced for eight years. She began Foxin Jing during a prolonged period of family tension and found regular participation in collective practice difficult because of domestic responsibilities. Rather than abandoning the practice when group meetings were unavailable, she developed a solitary routine during quieter periods at home:

“If there is no group practice opportunity, this may never happen. Practicing alone is easier for me.”

P7 did not experience solitary recitation as religious isolation. Instead, practicing alone allowed religious commitment to coexist with the demands of family life. She used Foxin Jing particularly when emotional pressure intensified, suggesting that the practice became responsive to the circumstances of her household rather than remaining tied to a predetermined ritual schedule. Her experience illustrates how adaptation was not peripheral to the practice but central to its continuity.

#### The case of P11

P11, a freelancer in her early thirties and one of the more recent practitioners in the sample, entered Foxin Jing through a female friend. She was not initially persuaded by formal instruction, doctrinal explanation, or institutional endorsement. What mattered was the transformation she believed she could observe in another practitioner:

“I learned Foxin Jing from a friend of mine who did the practice before me. Nothing fancy happened, but I believed it because I could see the difference in her.”

P11’s account reveals an important feature of lay transmission. The credibility of the practice was established through observation: greater calmness and emotional steadiness in another woman became evidence that Foxin Jing “worked.” She therefore learned the practice not only from what another practitioner told her but from how that practitioner appeared to have changed. Transmission here depended on interpersonal trust and observable conduct, showing how religious practice could circulate through relationships even without formal initiation or institutional certification.

#### The case of P1 and P9

For P1 and P9, Foxin Jing became particularly meaningful in relation to emotional pressure. P1, a housewife in her early forties with twelve years of practice, adjusted the frequency of recitation according to the intensity of problems at home:

“When I have a lot of problems at home, I recite more often. When things are calm, sometimes I only do it briefly.”

Her account shows that the practice did not operate according to a fixed devotional timetable; it intensified when emotional demands increased. P9, a trader in her late forties with approximately fifteen years of practice, described a more gradual transformation. She did not claim that anger or anxiety disappeared. Instead, she emphasized a change in the timing of her reaction:

“I used to panic and get angry easily. After reciting Foxin Jing for a long time, my reactions are slower, as if there is a gap between feelings and actions.”

The significance of this “gap” lies in the felt interval between emotion and response. For P9, sustained recitation did not eliminate difficult emotions but changed how quickly those emotions became action. She associated this delay with greater restraint in family and work-related situations. Together, P1 and P9 show how Foxin Jing became integrated into emotional life at two levels: as a situational practice activated during periods of pressure and as a longer-term discipline that altered the experience of emotional reactivity.

#### The case of P10

P10, a housewife in her early fifties with more than twenty years of practice, articulated this transformation in explicitly bodily terms. Rather than describing the efficacy of Foxin Jing through doctrinal knowledge, she recognized changes first through bodily tension:

“I knew my heart was starting to calm down because my body was no longer tense.”

Her account was consistent with field observations in which recitation was accompanied by slower breathing, reduced movement, softened facial expression, and extended periods of silence. Practitioners did not describe these bodily changes as incidental physical effects. They functioned as signs through which participants interpreted the condition of the heart-mind. For P10, knowing that the practice was having an effect did not begin with abstract reflection; it began with sensing that the body was different.

#### The case of P13 and P19

The accounts of P13 and P19, both long-term practitioners, add a temporal dimension to these experiences. P13, a retired woman with approximately twenty-five years of practice, summarized the change in meaning succinctly:

“At first, it was protection. Later, it became a way to look inside.”

In her account, “protection” referred broadly to maintaining family safety, coping with uncertainty and misfortune, and sustaining a sense of security during difficult periods. Over time, however, the practice also became associated with introspection and self-cultivation. P19, a retired woman who had practiced for approximately thirty years, similarly described Foxin Jing as a source of continuity amid wider social change:

“The world outside changes, but this practice gives me something steady.”

These accounts do not indicate a simple replacement of protection by self-cultivation. Rather, protective and reflective meanings accumulated across different stages of life. For senior practitioners, Foxin Jing remained capable of providing security during periods of uncertainty while also becoming a means of examining the self and maintaining inner stability.

Taken together, these cases show that becoming a Foxin Jing practitioner involves more than acquiring a ritual technique. Women entered the practice through interpersonal relationships, adapted it to domestic and occupational constraints, intensified it in response to emotional pressures, and evaluated its efficacy through changes in bodily sensation, emotional response, and the conduct of other practitioners. P3 and P7 demonstrate how the practice becomes sustainable within domestic life; P11 shows how observable transformation generates interpersonal trust; P1 and P9 reveal its integration into emotional experience; P10 demonstrates how bodily change becomes a marker of inner transformation; and P13 and P19 show how the meaning of the practice develops across long periods of religious life. Across these different trajectories, Foxin Jing emerges as a practice learned through relationship, sustained through adaptation, and understood through lived bodily and affective experience.

## Discussion

The first contribution of this study is to reposition the domestic sphere from a residual location of religious practice to an active infrastructure of esoteric continuity. The findings show that Foxin Jing does not depend exclusively on temples, monastic schedules, or formally organized ritual communities to remain religiously meaningful. This pattern resonates with lived-religion scholarship, which has demonstrated that religion is continuously produced through ordinary routines, relationships, material settings, and practical negotiations rather than solely through institutional structures (McGuire, 2008; Ammerman, 2014). In the Chinese Buddhist context, however, the present findings extend this argument by showing that domesticization does not necessarily imply the dilution or privatization of esoteric practice. Instead, the home enables Foxin Jing to remain ritually repeatable while accommodating women’s occupational, familial, and temporal constraints. Domestic space therefore functions not merely as the location in which an institutional practice is reproduced, but as a social environment through which esoteric practice is reorganized around the rhythms of lay women’s everyday lives.

A second implication concerns the relationship between formal lineage and everyday religious transmission. Esoteric Buddhism conventionally places considerable weight on teachers, initiation, ritual authorization, and lineage continuity (Bentor and Shahar, 2017; Larson, 2020; Proffitt, 2023), and these structures remain important in the Heart-of-Mind tradition. Yet the present study demonstrates that the continuation of practice after transmission depends on a wider relational ecology. Women encounter, evaluate, repeat, and sometimes introduce Foxin Jing to others through friendship, family relations, observation, and perceived changes in fellow practitioners. This does not replace vertical guru-disciple authority; rather, it reveals a horizontal layer through which formally transmitted practice becomes socially durable. In this respect, the findings complement studies showing that Buddhist networks are sustained through relationships extending beyond formal religious offices (Péronnet, 2021, 2022; Lau, 2022). The distinction is important: lineage authorizes the practice, whereas everyday relational networks help make the practice inhabitable and transmissible. Religious transmission should therefore be understood not only genealogically but also relationally and experientially.

The most theoretically consequential finding concerns the bodily and affective basis through which participants recognized the efficacy of Foxin Jing. Participants frequently described change not through doctrinal mastery but through slower breathing, reduced bodily tension, emotional steadiness, and an increased interval between affective stimulus and reaction. This finding is broadly compatible with embodied-religion approaches, which locate religious experience in bodily performance, sensory perception, gesture, voice, and affect rather than treating the body as a passive carrier of belief (Csordas, 2002; McGuire, 2008). It also intersects with lived religion and scholarship on ritual embodiment. However, these frameworks primarily explain how religion is experienced through the body. The present study raises a further analytical question: when does embodied experience itself become a source of religious credibility? In the Foxin Jing cases, bodily and affective transformation was not merely an outcome of practice; it also became evidence through which practitioners judged the practice as effective and through which others came to trust those who practiced it. This distinction provides the empirical basis for the concept of somatic authority developed in this article.

Somatic authority, as used here, refers to the situated religious credibility generated when sustained practice produces bodily and affective changes that practitioners and socially legible to others recognize. It differs from institutional authority because it does not derive from office, ordination, or organizational position; from lineage authority because it does not itself authorize esoteric transmission; and from embodied religion because its analytical focus is not simply embodiment but the conversion of embodied transformation into credibility. Nor is it equivalent to emotional regulation: emotional steadiness is one possible manifestation through which such authority becomes perceptible, rather than the concept itself. Somatic authority therefore helps explain why lay women who possess little formal religious power may nevertheless become trusted reference points within everyday practice networks. Importantly, this argument does not imply that embodied experience displaces teachers or lineage. Rather, it identifies a secondary and relational form of authority operating within the boundaries of established esoteric structures.

Taken together, these findings complicate interpretations of women’s Buddhist agency that privilege either institutional advancement or explicit resistance to religious hierarchy. Studies of Chinese Buddhist women have demonstrated both persistent structural asymmetries and women’s capacity to negotiate access, networks, practice opportunities, and religious influence within them (Péronnet, 2021, 2022; Lau, 2022). The Foxin Jing cases reveal a related but analytically distinct form of agency. These women do not generally challenge guru authority, claim formal ritual leadership, or seek to replace institutional structures; instead, they exercise agency by reorganizing when and where practice occurs, sustaining relational networks, incorporating recitation into domestic and occupational life, and cultivating forms of embodied credibility. This suggests that post-reform Chinese Buddhist modernity should be examined not only through state regulation, institutional revival, temple reconstruction, and formal religious organizations, but also through the mundane religious labour by which practitioners make traditions sustainable under changing conditions of family life, employment, mobility, and emotional pressure. The endurance of Foxin Jing therefore demonstrates that religious continuity can occur through adaptation without requiring either institutional expansion or doctrinal transformation.

## Conclusion

The findings demonstrate that lay women in the Heart-of-Mind Esoteric Buddhist tradition contribute to the continuity of Foxin Jing not primarily through formal religious office, but through practices embedded in domestic life, interpersonal relationships, and embodied experience. Across the twenty participants, Foxin Jing was sustained through flexible household routines and informal lay networks. At the same time, its efficacy was interpreted through bodily relaxation, emotional composure, slower reactivity, and long-term changes in the meaning of practice. These findings extend analyses of contemporary Chinese Buddhism beyond institutional representation by showing how esoteric traditions remain socially durable within ordinary lay life. Most importantly, the study develops the concept of somatic authority to explain how embodied and affective transformation can become socially recognizable as evidence of religious efficacy and a basis for interpersonal trust. Somatic authority does not replace lineage, guru authority, or institutional legitimacy; rather, it identifies a relational form of credibility through which lay women contribute to religious transmission from within established esoteric structures. The case of Foxin Jing therefore shows that the continuity of contemporary Chinese esoteric Buddhism depends not only on institutional preservation, but also on the capacity of practitioners to make religious practice repeatable, credible, and meaningful within everyday life.

## Data Availability

The data supporting the findings of this study are available from the corresponding author upon reasonable request, subject to privacy and ethical restrictions concerning the research participants.
